# Practical guidance for the implementation of the CRISPR genome editing tool in filamentous fungi

**DOI:** 10.1186/s40694-019-0079-4

**Published:** 2019-10-17

**Authors:** Min Jin Kwon, Tabea Schütze, Sebastian Spohner, Stefan Haefner, Vera Meyer

**Affiliations:** 1 Chair of Applied and Molecular Microbiology, Institute of Biotechnology, Technische Universität Berlin, 10263 Berlin, Germany; 2 BASF SE, Carl-Bosch-Strasse 38, 67056 Ludwigshafen, Germany

**Keywords:** Filamentous fungi, Cell factory, *Thermothelomyces thermophilus*, *Myceliophthora thermophila*, CRISPR, Genome editing, Cas9, Cpf1, Cas12a, RNP, Multiplexing, Selection-free gene targeting

## Abstract

**Background:**

Within the last years, numerous reports described successful application of the CRISPR nucleases Cas9 and Cpf1 for genome editing in filamentous fungi. However, still a lot of efforts are invested to develop and improve protocols for the fungus and genes of interest with respect to applicability, scalability and targeting efficiencies. These efforts are often hampered by the fact that—although many different protocols are available—none have systematically analysed and compared different CRISPR nucleases and different application procedures thereof for the efficiency of single- and multiplex-targeting approaches in the same fungus.

**Results:**

We present here data for successful genome editing in the cell factory *Thermothelomyces thermophilus*, formerly known as *Myceliophthora thermophila*, using the three different nucleases SpCas9, FnCpf1, AsCpf1 guided to four different gene targets of our interest. These included a polyketide synthase (*pks4.2*), an alkaline protease (*alp1*), a SNARE protein (*snc1*) and a potential transcription factor (*ptf1*). For all four genes, guide RNAs were developed which enabled successful single-targeting and multiplex-targeting. CRISPR nucleases were either delivered to *T. thermophilus* on plasmids or preassembled with in vitro transcribed gRNA to form ribonucleoproteins (RNPs). We also evaluated the efficiency of single oligonucleotides for site-directed mutagenesis. Finally, we were able to scale down the transformation protocol to microtiter plate format which generated high numbers of positive transformants and will thus pave the way for future high-throughput investigations.

**Conclusion:**

We provide here the first comprehensive analysis and evaluation of different CRISPR approaches for a filamentous fungus. All approaches followed enabled successful genome editing in *T. thermophilus*; however, with different success rates. In addition, we show that the success rate depends on the respective nuclease and on the targeted gene locus. We finally present a practical guidance for experimental considerations aiming to guide the reader for successful implementation of CRISPR technology for other fungi.

## Background

Six million fungal species are estimated to exist on Earth [1], but we know only about 100,000 [2]. Most are saprophytes; however, many pose a threat for other organisms including man. Only a few are exploited in biotechnology as cell factories. *Aspergillus niger* has been the pioneer fungus of modern biotechnology and used for exactly 100 years for the production of citric acid and since then together with other fungal cell factories for many other products including organic acids, enzymes, drugs, antibiotics and vitamins to name but a few [3, 4]. To improve our understanding on fungal biology underlying pathogenicity or metabolic capabilities, fast and efficient genetic manipulation tools are a fundamental prerequisite.

The discovery of the CRISPR/Cas9 immune system of bacteria and archaea and their repurposing for genome editing has elicited a new era in genetic engineering for filamentous fungi. Successful applications have been reported for fungal cell factories since 2015 [5] including among others *Aspergillus niger*, *Penicillium chrysogenum*, *Trichoderma reesei* and *Thermothelomyces thermophilus*, which has recently been reviewed [6–8]. Notably, the Cas12a (Cpf1) system has recently been introduced as an alternative CRISPR tool for *A. niger* [9]. Compared to Cas9, Cpf1 recognizes T-rich PAM sequences and does not need a trans-acting crRNA (tracrRNA) due to the unique dual nuclease activity that cleaves not only the target DNA but also its own CRISPR-RNA (crRNA) [10]. Therefore, it is considered an improved alternative to Cas9 [11]. Currently, various CRISPR protocols have been published for filamentous fungal model strains and cell factories (Table 1), which reflect a broad applicability of this tool but considerably challenge the experimenter to choose the optimal methodology for a specific application or research question. So far, no structured survey has been performed, which systematically analyses and compares the efficiency of different CRISPR nucleases for single- and multiplex-targeting in a filamentous fungus and which could provide researchers a guidance outlining the advantages or disadvantages of different CRISPR approaches.

**Table 1:** Filamentous fungal model strains and cell factories for which CRISPR gene editing tools have been established. For details, see reviews [6–8]

Species	CRISPR protein	Nuclease delivery	References
*Aspergillus fumigatus*	Cas9	Plasmid-based and RNP-based	[36, 37]
*A. nidulans*	Cas9, Cpf1	Plasmid-based	[9]
*A. niger*	Cas9, Cpf1	Plasmid-based and RNP-based	[5, 9]
*A. oryzae*	Cas9	Plasmid-based	[38]
*Neurospora crassa*	Cas9	Plasmid-based	[39]
*Penicillium chrysogenum*	Cas9	Plasmid- based and RNP-based	[33]
*Thermothelomyces thermophilus*	Cas9	Plasmid-based	[12]
*Trichoderma reesei*	Cas9	Plasmid-based	[40]

Note that the nuclease can be delivered to the cell either plasmid encoded or as a purified protein, which has to be preassembled with in vitro transcribed gRNA to form ribonucleoproteins (RNPs)

To overcome this issue, we tested in the current study the efficiency of three different nucleases for gene targeting in the cell factory *T. thermophilus*. This filamentous fungus is of current research interest because it exhibits a large capacity for plant biomass degradation and represents a potential reservoir of novel enzymes for many industrial applications. It was formerly known as *Myceliophthora thermophila* and a CRISPR method based on Cas9 has been published for this cell factory in 2017 [12]. The market for enzymes is huge with a total value of approximately $ 4 billion in 2018 [13]. The market leader was Novozymes, with a share of 48%, followed by Danisco (21%), DSM (6%), AB Enzymes (5%) and BASF (4%). Within this market, household care enzymes made up 32% of sales, closely followed by food and beverage enzymes (29%), bioenergy (19%), agricultural and feed (14%) and other technical and pharma enzymes (6%). The *T. thermophilus* strain ATCC 42464 is predominantly used in academic research groups as the general wild-type strain. For industrial use, the proprietary mature enzyme production strain C1 was developed [14]. The main features of strain C1 are production levels up to 100 g/L protein, and the maintenance of low viscosity levels during fermentation.

We present here a comprehensive survey of different CRISPR gene-targeting approaches for the *T. thermophilus* strain ATCC 42464 including the successful implementation of two new Cpf1 nucleases. We tested the Cpf1 nucleases from *Francisella novicida* (FnCpf1) and *Acidaminococcus* sp. (AsCpf1) to broaden the genome editing toolbox and compared their performance to the well-established Cas9 nuclease from *Streptococcus pyogenes* (SpCas9). Note that the recognition sequence for FnCpf1 is 5′‐TTN‐3′ and 5′‐TTTN‐3′ for AsCpf1, whereas SpCas9 recognizes 5′‐NGG‐3′ [15]. Previous studies have shown that the genome editing efficiency can be different between AsCpf1 and FnCpf1. AsCpf1 performed better in human cell lines [16], whereas genome editing with FnCpf1 was more efficient in *S. cerevisiae* [17]. Single, double, triple and quadruple gene-targeting were successfully established in *T. thermophilus* and the efficiency of a plasmid-based or RNP-based provision of the respective nucleases compared. We finally optimized transformation protocols for both approaches with respect to efficiency and scalability.

## Results and discussion

### RNP application of FnCpf1, AsCpf1 and SpCas9 for single targeting

Strains of filamentous fungi that are deficient in the non-homologous end joining (NHEJ) pathway, i.e. with reduced ectopic integration events during transformation, are preferred as hosts for efficient genome editing due to their high frequencies of DNA integration via homologous recombination [18]. In the case of *T. thermophilus*, this was recently proven for the *ku70* gene, which is a central element of the NHEJ machinery. Its inactivation resulted in a threefold higher homologous recombination rate [12]. Therefore, we have deleted another central element of the NHEJ machinery, the predicted *ku80* ortholog, (MYTH_2118116), in the wild-type *T. thermophilus* strain ATCC42464 using SpCas9 and *amdS* as selection marker (for details see “Methods”). Correct deletion of *ku80* was verified in strain MJK19.4 by diagnostic PCR and Southern blot analysis (Additional file 1 and data not shown). This strain was selected for removal of the *amdS* gene via FAA counterselection (see “Methods”) resulting in strain MJK20.2.

**Fig. 1:**
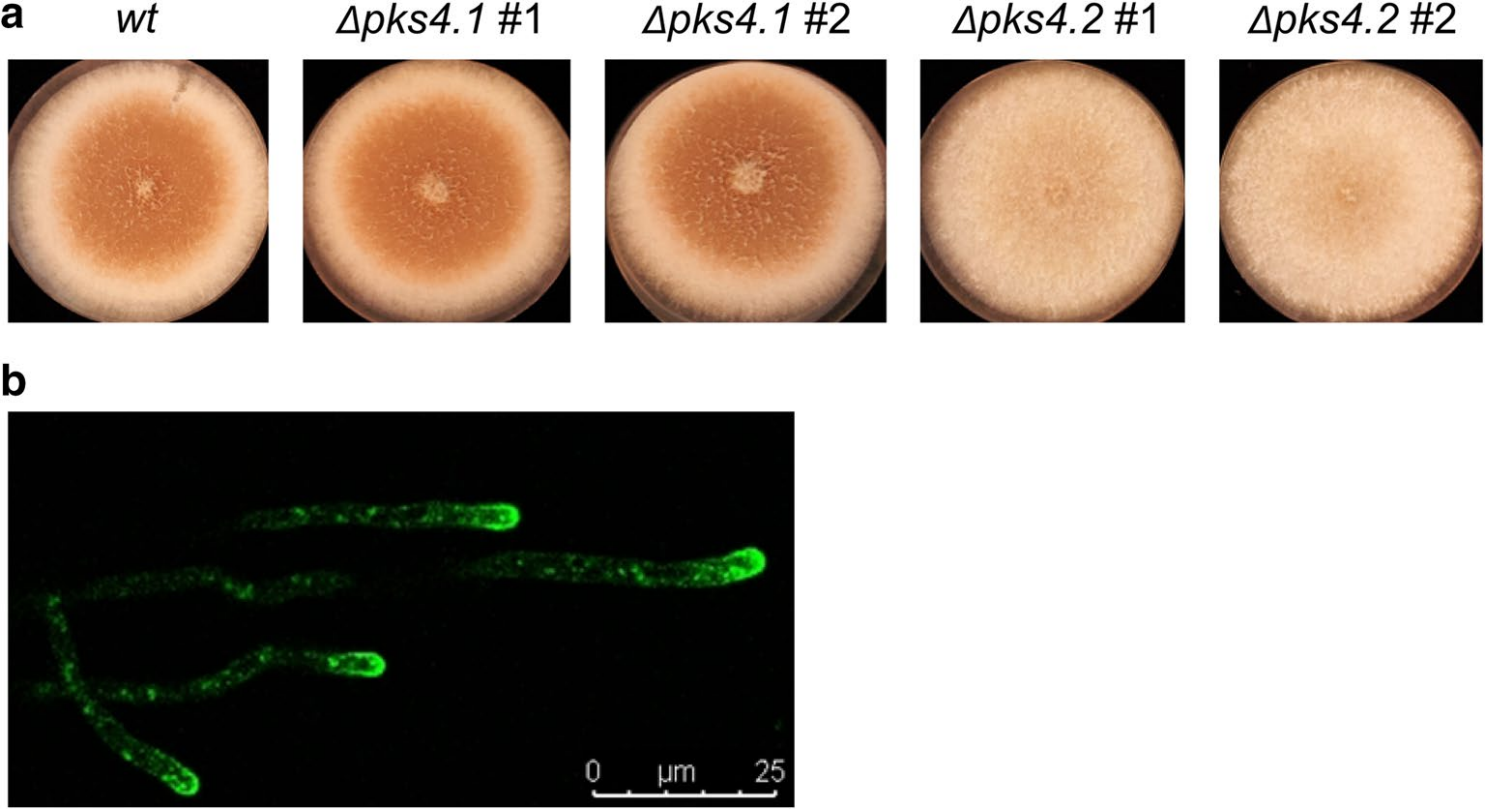
Phenotypes of *T. thermophilus* strains generated with CRISPR. **a** Deletion-phenotype of *pks4.1* and *pks4.2* strains cultivated on CM agar plates for 4 days at 37 °C. **b** GFP::Snc1 strains cultivated on MM agar for 12–16 h at 28 °C and analysed via fluorescence microscopy. Secretory vesicles accumulate at the hyphal apex. Note that hyphae of *T. thermophilus* do not autofluoresce under the experimental condition used (see “Methods”, data not shown)

In order to compare the three different CRISPR nucleases FnCpf1, AsCpf1 and SpCas9 regarding their targeting efficiency, we chose the *pks4*.2 gene and re-used the *amdS* gene for selection. In the *T. thermophilus* genome, two orthologs of the *pks4* gene described in *T. reesei* [19] are present, which we named *pks4.1* (MYTH_105482) and *pks4.2* (MYTH_2300170), respectively. However, only deletion of *pks4.2* results in an easy detectable spore colour change in *T. thermophilus*, thus allowing fast screening of potentially successful CRISPR mutants (Fig. 1a). RNP application, meaning the individual transformation of the respective pre-assembled RNP, of all three CRISPR nucleases achieved 100% targeting efficiency as confirmed by phenotypic and diagnostic PCR screenings (see “Methods”) and increased the transformation efficiency specifically for the FnCpf1 nuclease (Table 2). One has to note, however, that PAM sites are different for Cpf1 (TTN) and Cas9 (NGG); hence, different DNA motifs are targeted in the same gene of interest. This makes a direct comparison of targeting efficiencies difficult, as anything from base pairing of the gRNA, to the target sequence, to chromatin accessibility of the target site can affect this process. Nevertheless, it is assumed that NHEJ-mediated knock-in mediated by the annealing of cohesive ends might be facilitated using Cpf1 proteins, which produce cohesive ends with 4- or 5-nt overhangs, while SpCas9 produces blunt ends [20].

**Table 2:** Transformants and gene deletion efficiency targeting the *pks4.2* gene

Nuclease	No. of colonies analysed	Editing efficiency (%)
–	9	25
FnCpf1	43	100
AsCpf1	15	100
SpCas9	11	100

Phenotypic screens and diagnostic PCR for the *pks4.2* locus were performed as described in “Methods” section

### RNP application of FnCpf1, AsCpf1 and SpCas9 for multiplex-targeting

To investigate whether the three CRISPR nucleases support targeting of up to four genes simultaneously, three of which via a selection-free process, we performed the following strategy: (i) deletion of the *pks4*.2 gene (using *amdS* as selection marker) resulting in an easy detectable colour mutant (Fig. 1a), (ii) replacement of the endogenous *snc1* gene (MYTH_64173) with a functional *snc1::eGFP* fusion construct for detection of GFP fluorescence via confocal microscopy (note that *snc1* encodes a SNARE protein and is an established marker for secretory vesicles in filamentous fungi (Fig. 1b, [21]), (iii) deletion of the *alp1* gene (MYTH_2303011) encoding an alkaline protease which was previously shown to become successfully targeted by SpCas9 in *T. thermophilus* [12], and (iv) deletion of a non-verified protein encoding a predicted transcription factor. For brevity, we named it *ptf1* in this study. Donor DNAs were provided for all genes and details can be found in “Methods” section.

As depicted in Table 3, the targeting efficiencies of FnCpf1 and AsCpf1 are very similar. However, all three nucleases target the four gene loci with different efficiencies. Whereas the *ptf1* gene seems to be generally difficult to edit for all three nucleases, the *alp1* gene might only be difficult as a target for the SpCas9 nuclease, implying that the performance of CRISPR nucleases can also be locus-dependent. Alternatively, the same gRNA might be recognized with different efficiencies by the different nucleases, as shown in other studies (e.g. [22]). Surprisingly, SpCas9 was not able to target the *snc1* gene during two independent transformation attempts, although the respective in vitro control cleavage assay proved that the enzyme is functional with the gRNA provided (Additional file 2).

**Table 3:** Transformants and PCR-confirmed editing efficiency for the simultaneously targeted *alp1, pks4.2, snc1, and ptf1* gene loci

Nuclease	No. of colonies	Editing efficiency (%)			
		*alp1*	*pks4.2*	*snc1*	*ptf1*
–	1	0	100	0	0
FnCpf1	56	91	36	52	6
AsCpf1	80	92	10	49	0
SpCas9	40	20	97	0	20

Diagnostic PCR for all individual loci were performed as described in “Methods” section

**Fig. 2:**
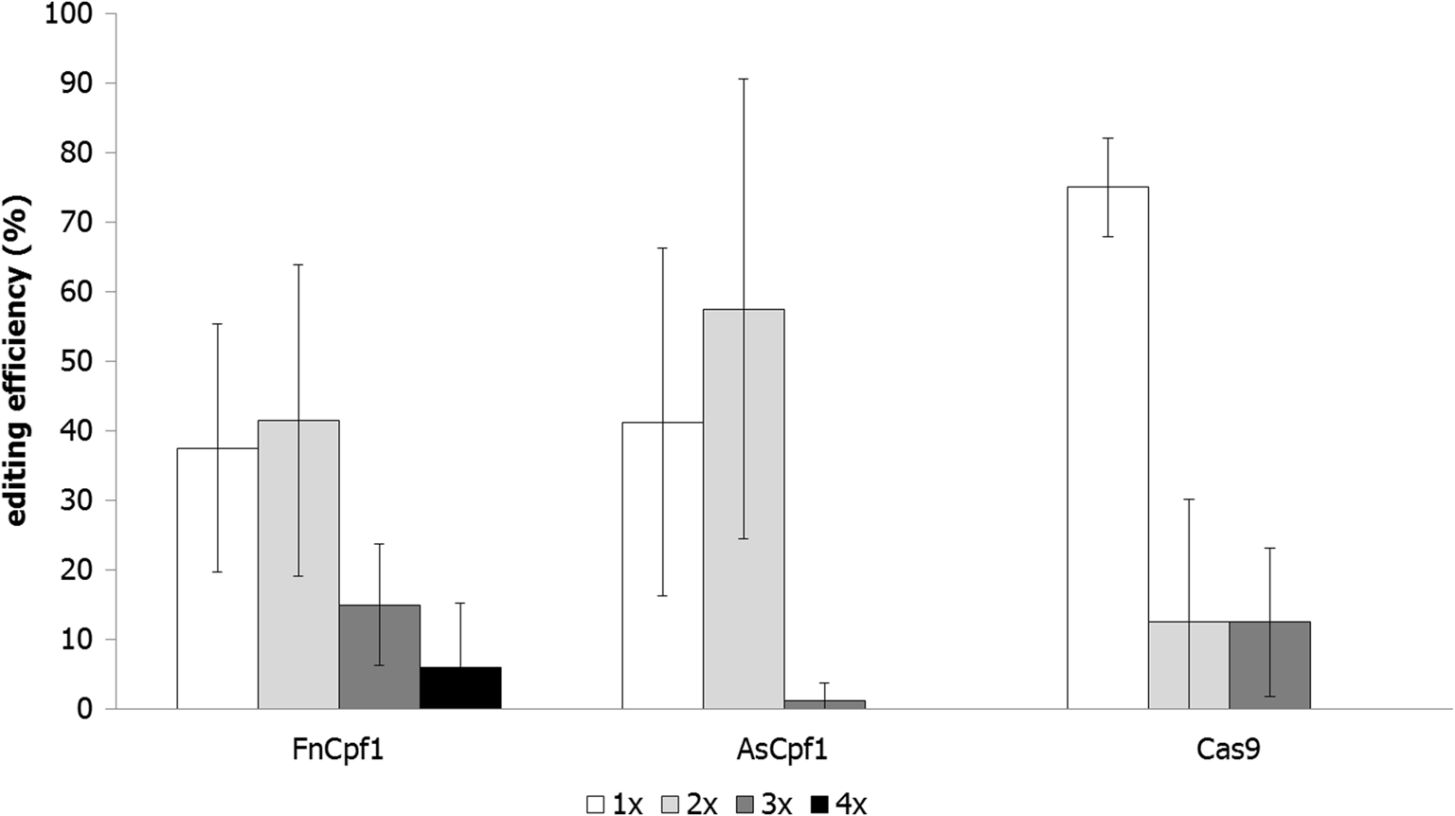
Editing efficiencies of four targets using the RNP approach. Three different nucleases were used to target 4 genes simultaneously (*pks4.2, alp1*, *snc1*, *ptf1*). Only the donor DNA of *Δpks4.2* contained the selection marker *amdS*. Up to four individual transformation experiments were performed and 40–80 transformants were analyzed for each setup

Notably, the identification of single, double, triple or quadruple targeting events in *T. thermophilus* induced by the RNP multiplexing approach uncovered that all nucleases display different abilities to target multiple genes simultaneously, whereby quadruple targeting events were very seldom(Fig. 2). Reduced targeting efficiency is a generally observed phenomenon when multiple genes become simultaneously targeted, as e.g. recently shown for the cell factory *A. niger* [23]. Interestingly, quadruple targeting events in *T. thermophilus* were only detectable for FnCpf1. We have therefore selected this nuclease for a comparative analysis with a plasmid-based expression of FnCpf1.

### Comparison of RNP-based and plasmid-based application of FnCpf1 for multiplex-targeting

**Fig. 3:**
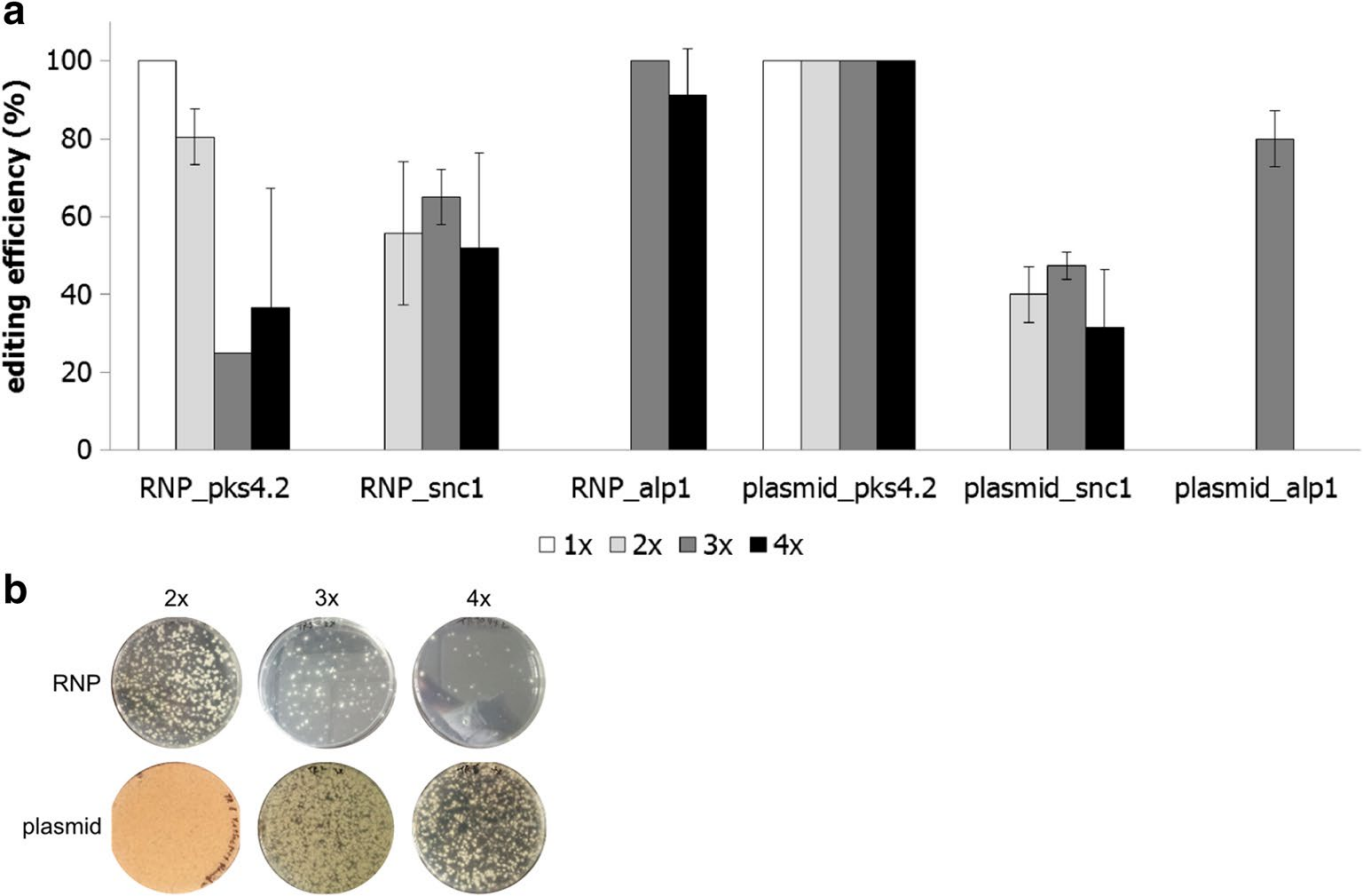
Comparison of the RNP- and plasmid-based approach. **a** Editing efficiencies for single, double, triple and quadruple targeting. FnCpf1 was used to target all 4 genes simultaneously (‘4×’ *pks4.2, alp1*, *snc1*, *ptf1*) or only double or triple combinations thereof. Up to ten individual transformation experiments were performed for each setup. Note that no experiments for RNP_snc1 (1×), RNP_alp1 (1×) and (2×), plasmid_snc1 (1×), plasmid_alp1 (1×), (2×) and (4×) were done. Data for *ptf1* were omitted in the diagram due to low targeting efficiency. **b** Pictures of transformation plates using different amounts of targets. Note that the targets for the RNP- and plasmid-based approach were identical except for 4× targeting. There, the targets were *alp1*, *pks4.2*, *snc1*, and *ptf1* for the RNP approach and *alp1*, *pks4.1*, *pks4.2* and *snc1* for the plasmid approach

For this study, we cloned the FnCpf1 encoding gene into one plasmid (MT2286) and used it for co-transforming it together with plasmids encoding all four gRNAs separated by direct repeats (plasmid pMJK31.1 for *pks4.2* and *snc1*, plasmid pMJK32.2 for *alp1* and plasmid pMJK33.1 for *pks4.1* and *ptf1*) and each respective donor construct for the *pks4.2, snc1, alp1 and ptf1* genes into *T. thermophilus* (see “Methods”). All plasmids ensured transient expression of the *fncpf1* gene and the gRNA encoding sequences. This setup was used for direct comparison with RNP-based application of FnCpf1, which was performed in parallel. Note that for these experiments an improved transformation protocol was developed, in which the transformation efficiency was considerably increased by using PEG-4000 instead of PEG-6000 (see “Methods”). As summarized in Table 4, some differences were observed between single targeting when the FnCpf1 nuclease was provided via RNPs or on a plasmid to *T. thermophilus* (*alp1* p = 0.06, *pks4.2* p = 0.04, *ptf1* p = 0.83, *snc1* p = 0.02, p-values were calculated using a two-tailed students *t* test). However, the efficiency of double, triple and quadruple targeting considerably improved when the *fncpf1* was transiently expressed from a plasmid (Fig. 3a). Remarkably, the overall transformation frequency was higher in the plasmid-based approach compared to the RNP approach (Fig. 3b). This data might suggest that plasmids might be easier taken up by protoplasts compared to RNPs and/or that both FnCpf1 and gRNAs might be more abundant or longer available intracellularly when transiently expressed. For both cases, however, the transformation frequency decreased considerably with an increase in target loci (Fig. 3b).

**Table 4:** Editing efficiency for four different gene loci of FnCpf1

Target	RNP				Plasmid			
	Mean [%]	SD [%]	n	No. of analysed transformants	Mean [%]	SD [%]	n	No. of analysed transformants
*alp1*	93.9	9.4	7	108	80	7.1	4	78
*pks4.2*	50	34.3	13	228	100	0	10	158
*snc1*	57.2	17.7	11	185	40	10.4	6	118
*ptf1*	7	6.6	3	28	5.5	7.8	2	38

*SD* standard deviation, *n* number of transformations performed

Diagnostic PCR was done for *alp1* and *ptf1* loci as described in “Methods” section. For *snc1,* fluorescence microscopy and for *pks4.2,* the colour of conidia was used to verify successful integration of the donor DNA. The donor DNA for *pks4.2* was carrying the *amdS* selection marker

### SON-based targeting of FnCpf1 and SpCas9

Single-stranded oligonucleotides (SONs) have been shown to be efficient templates for the repair of SpCas9 and LbCpf1 (from *Lachnospiraceae bacterium*) induced DNA double-strand breaks in NHEJ-deficient *A. nidulans* and *A. niger* [9, 24]. We therefore tested whether this approach (which can be harnessed to introduce specific point mutations into the locus of interest) can also be followed using FnCpf1 and SpCas9 nucleases in *T. thermophilus*. We thus applied 90 bp long oligonucleotides homologous to part of the *pks4.2* locus which were designed to introduce three stop codons in the centre part (Additional file 3). The selection marker was present on the donor DNA for the second target, *pks4.1* (plasmid pMJK22.19). In total, 30 (25) *amdS* expressing transformants were identified for FnCpf1 (SpCas9), 5 (3) of which displayed the respective spore colour change indicative for a *pks4.2* gene inactivation. All eight transformants were picked and sub-cultivated. Correct integration of the *pks4.1* donor DNA was verified by PCR and the respective *pks4.2* locus PCR amplified and sequenced. The sequencing results verified that all 8 transformants were successfully targeted by both nucleases and that the SON introduced the desired gene edits (Additional file 3). For the first time, this data provides evidence that an oligonucleotide-mediated repair approach can be followed in *T. thermophilus* for site-directed mutagenesis applying either FnCpf1 or SpCas9.

### MTP-based method for high-throughput gene targeting

**Fig. 4:**
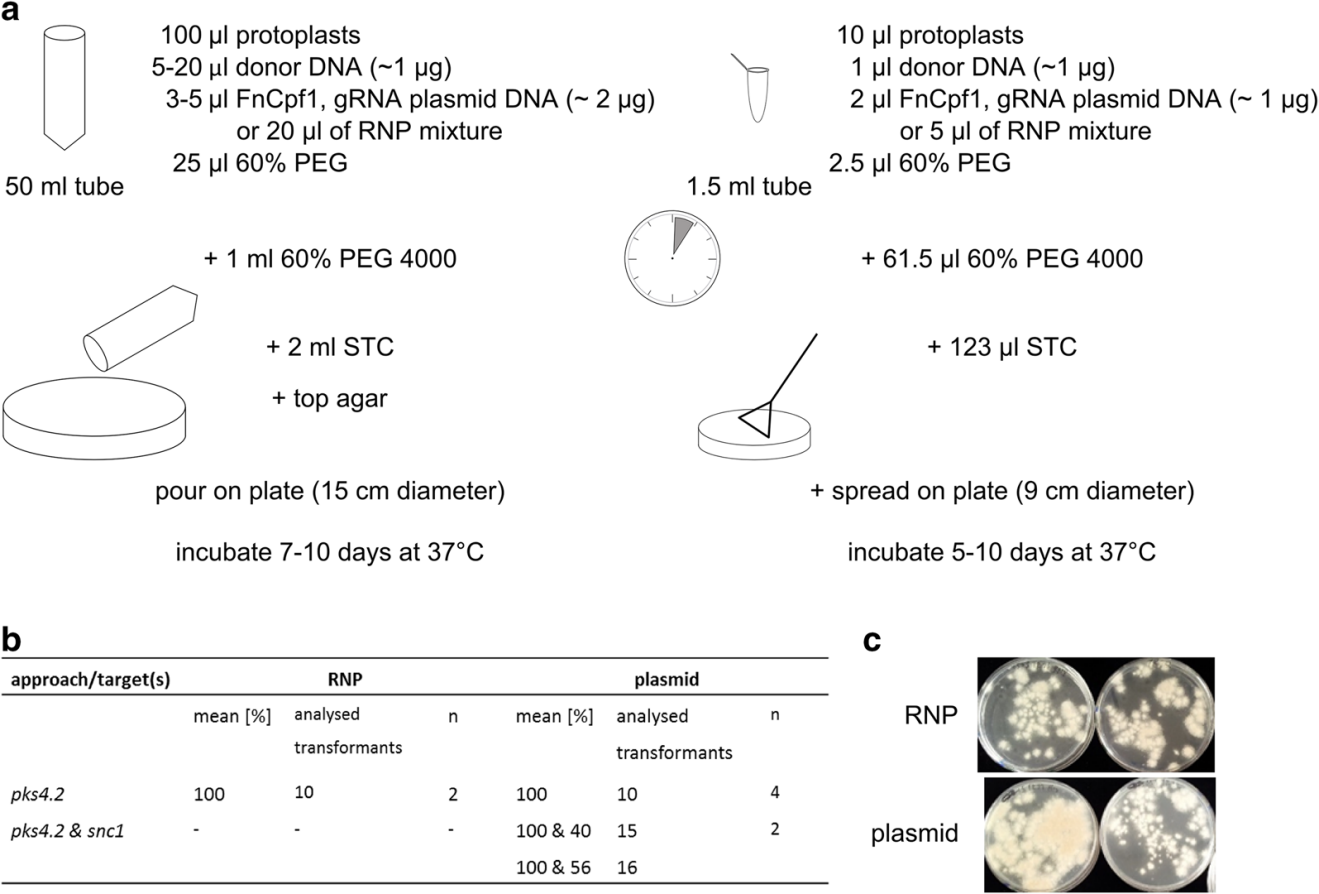
Downscaling the transformation procedure. **a** Comparison of medium- and small-scale transformation, details on the methodology can be found in “Methods” section. **b** Table comparing the genome editing efficiency of small-scale transformation between the RNP- and plasmid-based approach, n = number of transformations performed. **c** Backside of four transformation plates from small-scale transformations are shown, which demonstrate that a reduced number of transformants will be gained with the small-scale protocol when compared to the medium-scale protocol (see Fig. 3b)

We finally provide here a protocol for high-throughput gene targeting in *T. thermophilus* using a microtiter plate (MTP) compatible method. MTP-based approaches are fundamental for the development of cost-effective workflows used for genome-wide mutant libraries and high-throughput screenings for protein production. Recently, a respective protocol has been reported for *A. niger* [25], however, none has been published for *T. thermophilus* so far. Figure 4 summarizes the key aspects of this down-scaled approach, where 10 μL of protoplast solution (~ 5 × 10^5^ protoplasts) are sufficient to obtain > 30 transformants with either an RNP- or plasmid-based approach of FnCpf1 targeting the genes *pks4.2* and/or *snc1*, respectively. As with the classical transformation protocol, the single targeting efficiency was 100% (tested for *pks4.2* only) and the double targeting efficiency was 40–56% (tested for *pks4.2* and *snc1*). Similarly, plasmid-based application of FnCpf1 yielded more transformants than the RNP-based application on MTP scale. An MTP-based workflow is thus indeed very promising for high-throughput approaches. In this context, it is interesting to note that the addition of the cryoprotectant polyvinylpyrrolidone 40 to freshly harvested protoplasts from *T. thermophilus* allowed us to store protoplasts at − 80 °C for several weeks prior to transformation with no significant reduction in viability and transformability (see “Methods”, data not shown).

## Conclusions

CRISPR applications in fungal systems are on exponential rise with the same pace as in other biological systems and drive new research for medically and industrially relevant filamentous fungi [26]. Dozens of CRISPR articles have been published for filamentous fungi including many reviews since the first report on CRISPR–Cas9 application in Aspergilli in 2015 [5]. The huge scientific interest in the community to explore fungal diversity and specification even within a single genus [27, 28] will eventually lead to more filamentous fungi of interest for which efficient genetic transformation and genome editing tools have to be developed. The work presented here systematically investigated and evaluated different experimental approaches for the cell factory *T. thermophilus*, most of which were not tested yet for this fungus. We could successfully establish a variety of scalable protocols enabling genome editing by three different CRISPR nucleases for single- and multiplex-targeting approaches using plasmid-based or RNP-based delivery of the enzymes. We furthermore provide evidences that SON-mediated mutagenesis is functional in *T. thermophilus* for two CRISPR nucleases. Table 5 summarizes opportunities, limitations and critical aspects related to practicability and efficiency based on our observations made for *T. thermophilus*. It also reflects what has been observed so far in other eukaryotic systems (see references cited above). It could thus guide fungal scientists for future implementation of the CRISPR technology for their filamentous fungus of interest. In general, the availability of different Cas nucleases is of advantage. In case one enzyme fails to target a gene of interest, it is likely that another will do. The approach which is followed to deliver the Cas nuclease will also affect editing efficiencies. Be it that a plasmid-based approach might be easier to handle and is less prone to degradation compared to a RNP-based approach, be it because transient expression from plasmids (which are presumably longer stable intracellularly compared to RNPs) might ensure higher abundance and longer availability of the Cas enzymes and their gRNAs. It can also be speculated that targeting with RNPs (especially when multiplexing is intended) might exceed the limit of DNA/RNA which can be taken up by protoplasts. The canonical view is that an excess of DNA lowers transformation efficiency in filamentous fungi. Another important aspect worth considering, especially when using SpCas9, is the potential of introducing unintended off-target mutations [20]. The chances for hitting potential off-targets is likely high when both integration of the Cas9 nuclease in the genome and its constitutive expression is performed, lower when transient expression is adjusted but should be lowest, when an RNP-based approach is followed.

## Methods

### Microbial strains and cultivation conditions

Fungal strains used in this study are given in Additional file 4. Strain MJK20.2 was used as progenitor isolate as this strain is deficient in the non-homologous end joining pathway (*Δku80*), reducing ectopic integration events during transformation and thus enabling targeted integration [29]. Strains were grown at 37 °C in minimal medium (MM) or complete medium (CM), consisting of MM supplemented with 1% yeast extract and 0.5% casamino acids [18]. All bacterial plasmids were propagated in *Escherichia coli* DH5α using 100 µg/mL ampicillin or 50 µg/mL kanamycin for selection.

### Molecular techniques

All molecular techniques were performed according to standard procedures described previously [30]. *T. thermophilus* transformation and genomic DNA extraction were performed as described elsewhere [18]. When required, plates were supplemented with acetamide (15 mM) and caesium chloride (10 mM). Primers and plasmids used in this study are given in Additional files 5 and 6, respectively. All plasmids were sequenced and will be made available on reasonable request. Strain MJK20.2 (*Δku80*) was generated as follows: *ku80* was deleted in the wild type strain ATCC42464 with FnCpf1 or SpCas9 using PCR-amplified split marker fragments containing the *amdS* marker and about 1.2 kb flanks each for homologous integration. The 3′ split marker fragment contained the 5′ flank to mediate a fast removal of the *amdS* marker. The resulting strain MJK19.1 was sub-cultivated on FAA medium plates to obtain the marker-free *Δku80* strain MJK20.2. Strains were analysed by Southern blot analysis to verify correct integration of the fragments and removal of the marker gene (Additional file 1). For all other targets, donor DNA with flanks of about 1 kb length each were used. The amounts of donor DNA are specified in the RNP-based and plasmid-based approaches described below.

### Genome editing using RNP-based approach

The plasmid containing the expression cassette for SpCas9 (pET28a/Cas9-Cys) was obtained from addgene (#53261). *T. thermophilus* codon optimized FnCpf1 and AsCpf1 was cloned into plasmid pET28a giving plasmid pMJK16.1 and pMJK17.1, respectively. *E. coli* strain Rosetta™ 2(DE3)pLysS (Novagen) was freshly transformed with the respective expression plasmids. Four mL of TB medium (12 g/L tryptone, 24 g/L yeast extract, 5 g/L glycerol, 2.31 g/L KH_2_PO_4_, 12.54 g/L K_2_HPO_4_) plus 50 µg/mL kanamycin and 20 µg/mL chloramphenicol were inoculated from a single colony and incubated at 37 °C and 250 rpm overnight. 400 µL of these precultures were used to inoculate 40 mL of TB medium including antibiotics, which was incubated at 37 °C and 250 rpm until an optical density (OD_600_) of 5.0–8.0 was reached (approximately 5–7 h). Main cultures (1 L in 5 L Erlenmeyer flasks) with TB medium, autoinduction solution (5 g/L glycerol, 0.5 g/L glucose, 2 g/L α-lactose monohydrate) and the corresponding antibiotic(s) were inoculated with these 40 mL cultures to an OD_600_ of 0.1 and incubated in shake flasks at 37 °C and 160 rpm for 2 h. Afterwards, the temperature was decreased to 18 °C and the cells cultivated for at least 18 up to a maximum of 40 h. Proteins were purified as described previously [31] using Ni–NTA resin (Qiagen Germany).

Target sequences were selected in silico using Cas-Designer and Cas-OFFinder (http://www.rgenome.net/cas-offinder/) [32]. Respective gRNAs including PAM sites were generated as described before [33]. In brief, gRNAs were in vitro transcribed using the T7 promoter with an additional ATG at the front (ATGTAATACGACTCACTATAGG). For sequence information, see Additional file 5.

RNP assembly was done as described previously [33] with the following modifications. Prior to the transformation into fungal protoplasts, RNP complexes were assembled containing 30 µg CRISPR nuclease (5 µL), 2 µL 10 × Cas9 activity buffer, 1 µL gRNA and 12 µL of nuclease-free water in a 1.5 mL reaction tube. The mixture was incubated at 37 °C for 15 min to allow RNP complex formation. For multiplex-targeting, each target RNP complex was formed separately. For each transformation, 100 µL protoplasts, 10 µL donor DNA (5 µg), 20 µL RNP complex (up to 80 µL for multiplex-targeting), 20 µL 2× STC, 25 µL 60% PEG 4000 buffer and 20 µL 10 × Cas9 activity buffer were mixed in a 50 mL Greiner tube. Transformations with plasmid MT28 and/or sterile water served as controls. Note that this protocol differed from [18] with respect to PEG: 60% PEG 4000 was used in this study instead of 25% PEG 6000. Transformants were sub-cultivated twice on medium with 15 mM acetamide as nitrogen source. Genomic DNA was extracted from transformants. Insertion of the donor cassette at the respective locus was confirmed by diagnostic PCR.

### Genome editing using plasmid-based approach

3 µg of the *fncpf1* encoding plasmid MT2286 was co-transformed with 2 µg of each plasmid DNA encoding respective gRNAs separated by direct repeats (e.g. pMJK31.1 for *pks4.2* & *snc1* gRNA) as described by [34] and 3 μg donor DNA into *T. thermophilus* as follows: 100 µL protoplasts (~ 5 × 10^6^ protoplasts), 10 µL total DNA and 25 µL 60% PEG 4000 buffer were mixed in a 50 mL Greiner tube at room temperature. For transcription of the gRNA the U6 promoter was used. Expression of *fncpf1* was done according to [34, 12]. For codon optimisation the most frequent codons were used [35]. Transformations with plasmid MT28 and/or sterile water served as controls. Note that this protocol differed from [18] with respect to PEG: 60% PEG 4000 was used in this study instead of 25% PEG 6000. Transformants were sub-cultivated twice on medium with 15 mM acetamide as nitrogen source. Genomic DNA was extracted from putative transformants. Insertion of the donor cassette at the respective locus was confirmed by diagnostic PCR.

### Genome editing using SON-based approach

SON-based donor DNA targeting *pks4.2* was designed with 35/32 bp (up-/downstream) homologous arms containing 3 stop codons. For sequence information, see Additional file 3. Selection was based on the *Δpks4.1* deletion cassette (pMJK22.19), hence a double targeting approach was followed: RNP complexes were assembled containing 30 µg FnCpf1 (5 µL), 2 µL 10 × Cas9 activity buffer, 1 µl gRNA and 12 µL of nuclease-free water in a 1.5 mL reaction tube. The mixture was incubated at 37 °C for 15 min to allow RNP complex formation. For each transformation, 100 µL protoplasts, 5 µL donor DNA (5 µg), 10 μL SON (100 μM stock solution), 40 µL RNP complex, 20 µL 2× STC, 25 µL 60% PEG 4000 buffer and 20 µL 10 × Cas9 activity buffer were mixed in a 50 mL Greiner tube. Note that this protocol differed from [18] with respect to PEG: 60% PEG 4000 was used in this study instead of 25% PEG 6000. Transformants were sub-cultivated twice on medium with 15 mM acetamide as nitrogen source. Genomic DNA was extracted from transformants. Insertion of the donor cassette at the respective locus was confirmed by diagnostic PCR.

### Genome editing using MTP-based approach

The volume for the transformation reaction was reduced to 200 μL and transformation was done in a 1.5 mL reaction tube. Both, freshly prepared and cryopreserved protoplasts have been used. For one transformation using the plasmid approach, 10 μL protoplasts (~ 5 x 10^5^) were mixed with 1 μL donor DNA (1 μg), 1 µL FnCpf1 (1 μg), 1 μL gRNA plasmid (1 μg) and 2.5 μL 60% PEG 4000 buffer at room temperature. Afterwards 61.5 μL 60% PEG 4000 buffer was added and exactly five minutes later 123 µL STC was added. Instead of using top agar to distribute cells on an agar plate (15 cm diameter), the 200 μL protoplast mix was spread onto a small plate (9 cm diameter). For the RNP approach, 5 μL of RNP mixture was added from a 20 μL RNP complex reaction mixture. Identification and analysis of transformants were performed as described above.

### Cryopreservation of protoplasts

350–500 μL protoplasts (~ 1 × 10^7^) were mixed 1:1 with 20% Polyvinylpyrrolidone 40 solved in STC buffer. This mixture was deep frozen at − 80 °C using isopropanol for − 1 °C/min freezing. Before transformation, frozen protoplasts were washed with 10 mL cold STC buffer and spun down for 5 min at 1500 rpm and 4 °C. Protoplasts were resuspended with cold STC and used for transformation.

### In vitro SpCas9 cleavage assay

In brief, the plasmids with either the original Psnc1::gfp::snc1 sequence or the SpCas9 PAM site mutated sequence were used as donor DNAs. Each 600 ng were restricted with 10 U NotI in a total volume of 20 µL to generated linearized DNAs. After heat-inactivation (20 min at 80 °C) of *Not*I, the mixtures were immediately added without further purification to a 30 µL reaction mixture containing 1 µL gRNA and 1 µL Cas9 protein. After 60 min incubation at 37 °C, the reaction was quenched by adding 3 µL 0.5 M EDTA and 7 µL 6× gel loading dye. Samples were incubated for 15 min at 65 °C and analysed by electrophoresis on a 1% agarose gel.

### Genotypic, phenotypic and microscopic screens of CRISPR transformants on agar media

Putative transformants were analysed as follows: *Δpks4.2* transformants were sub-cultivated three times on MM agar medium and the spore colour formation compared to the wild-type strain. In case of questionable phenotypes, strains were further subjected to diagnostic PCR. Integration of Psnc1::gfp::snc1 was analysed using fluorescence microscopy. In brief, colonies cultivated on selective agar MM medium containing 15 mM acetamide for 24 h at 37 °C and fluorescence images were taken using an inverted TCS SP8 (Leica, Germany) as described earlier [30]. Most colonies with GFP-secretory vesicle signals had correct integration of the donor DNA at the *snc1* locus as checked by diagnostic PCR (~ 99%). Consequently, transformants with GFP- secretory vesicle signals, were considered *snc1* targeted. For *Δpks4.1*, *Δalp1* and *Δptf1* diagnostic PCR was done on the corresponding locus. For primer sequence information, see Additional file 5.

## Supplementary information

**Additional file 1.** Verification of *ku80* deletion and marker removal.

**Additional file 2.** In vitro cleavage assay of *snc1* target site for SpCas9.

**Additional file 3.** Application of SNOs using Cpf1 and Cas9.

**Additional file 4.** Strains used in this study.

**Additional file 5.** Primers and gRNA sequences used in this study.

**Additional file 6.** Plasmids used in this study.

## Data Availability

The data sets, strains used and/or analysed during the current study, and sequences are available from the corresponding authors on reasonable request.

## Abbreviations

CM: Complete medium
Cas: CRISPR-associated proteins
Cpf1: CRISPR from *Prevotella* and *Francisella*
CRISPR: Clustered Regularly Interspaced Short Palindromic Repeats
MM: Minimal medium
MTP: Microtiter plate
RNP: Ribonucleoprotein
SON: single-stranded oligonucleotide
