# Correction to: Practical guidance for the implementation of the CRISPR genome editing tool in filamentous fungi

**DOI:** 10.1186/s40694-019-0082-9

**Published:** 2019-11-13

**Authors:** Min Jin Kwon, Tabea Schütze, Sebastian Spohner, Stefan Haefner, Vera Meyer

**Affiliations:** 1 Chair of Applied and Molecular Microbiology, Institute of Biotechnology, Technische Universität Berlin, 10263 Berlin, Germany; 2 BASF SE, Carl-Bosch-Strasse 38, 67056 Ludwigshafen, Germany

## Correction to: Fungal Biol Biotechnol (2019) 6:15 10.1186/s40694-019-0079-4

Following publication of the original article [1], the authors reported that Table 5 was missing in the published version, although it was originally submitted and reviewed along with the rest of the manuscript. The complete Table 5 is given in this erratum.

**Table 5:** Practical guidance for the implementation of CRISPR technology in filamentous fungi based on data obtained for *T. thermophilus* in this study

	Plasmid-based approach	RNP-based approach
Preparation of nuclease	Cloning of the nuclease into a plasmid prior transformation is mandatory. When constitutively expressed, risk of off-targets should be considered. When present on AMA-plasmid, the risk should be lower but still present	Cloning of the nuclease into a plasmid allowing heterologous expression, e.g. in *E. coli*, is a prerequisite. Once established and purified, the nuclease can be aliquoted and stored prior to use. As the protein does not become expressed in the targeted fungus, the risk of off-targets should be very small
Preparation of guide RNA	Plasmid-based, thus more stable during handling and storage	Involves in vitro transcription, hence potentially sensitive to handling errors
Transformation procedure	Easy	Easy but requires preassembly of RNPs
Transformation rate	Very high also with four targets	Very high for single and double targets Low for three and four targets
Single-targeting efficiency of FnCpf1, AsCpf1, SpCas9	Locus-dependent	Locus-dependent
Multiplex-targeting efficiency of FnCpf1	High (34 ± 6% in this study)	Low (13 ± 2% in this study)
MTP-based down-scaling for FnCpf1	Possible with no loss in efficiency with respect to single and double targeting	Possible with no loss in efficiency with respect to single targeting^a^

^a^Double targeting was not tested

The original article has been corrected.
