# Supplementary figures and images for: Practical guidance for the implementation of the CRISPR genome editing tool in filamentous fungi
Source: Fungal Biol Biotechnol. 2019 Oct 17;6:15. doi: 10.1186/s40694-019-0079-4 (PMC6796461; doi:10.1186/s40694-019-0079-4)

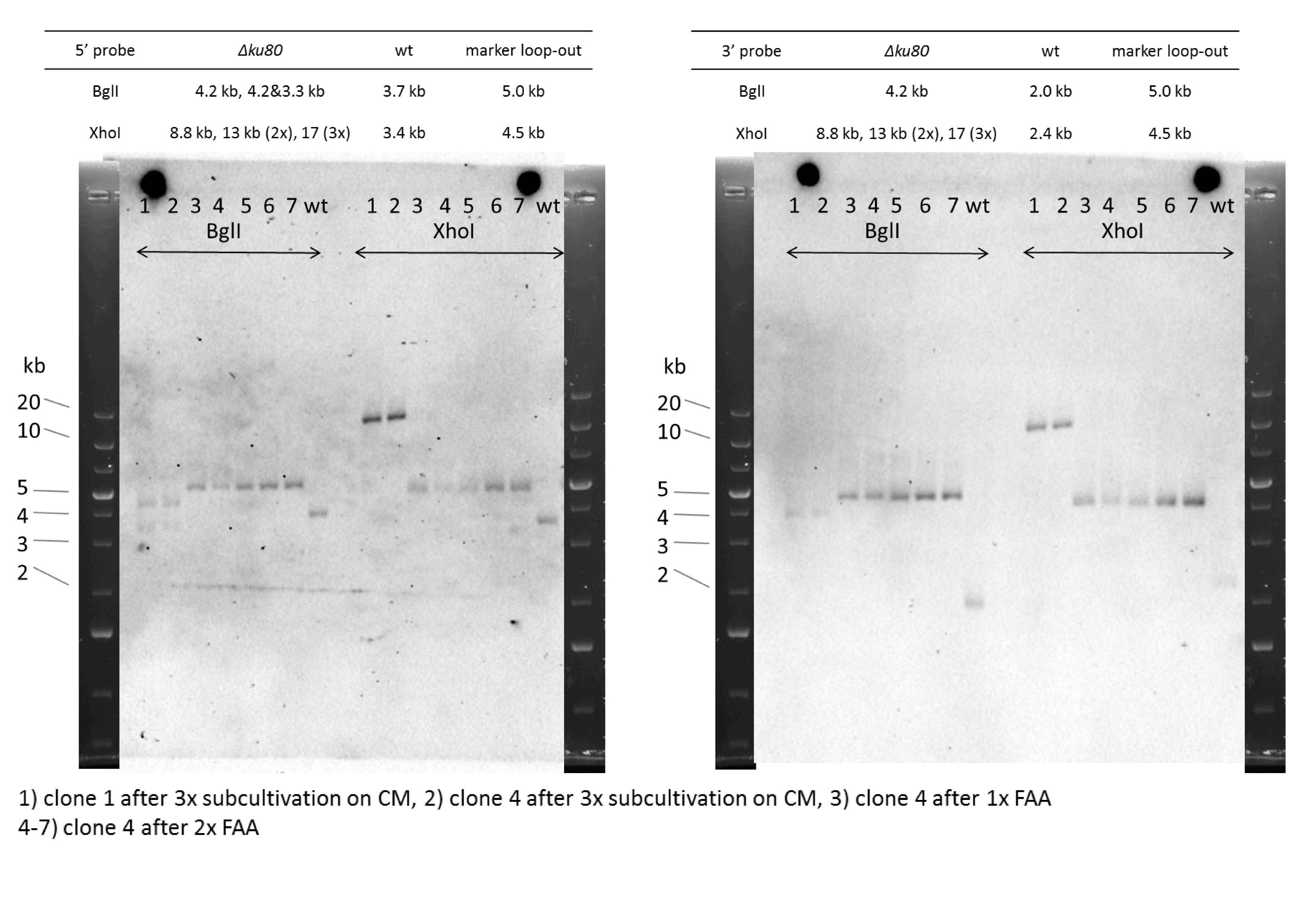

Supplement: Supplementary file 1 — Additional file 1. Verification of ku80 deletion and marker removal. [file 40694_2019_79_MOESM1_ESM.png]

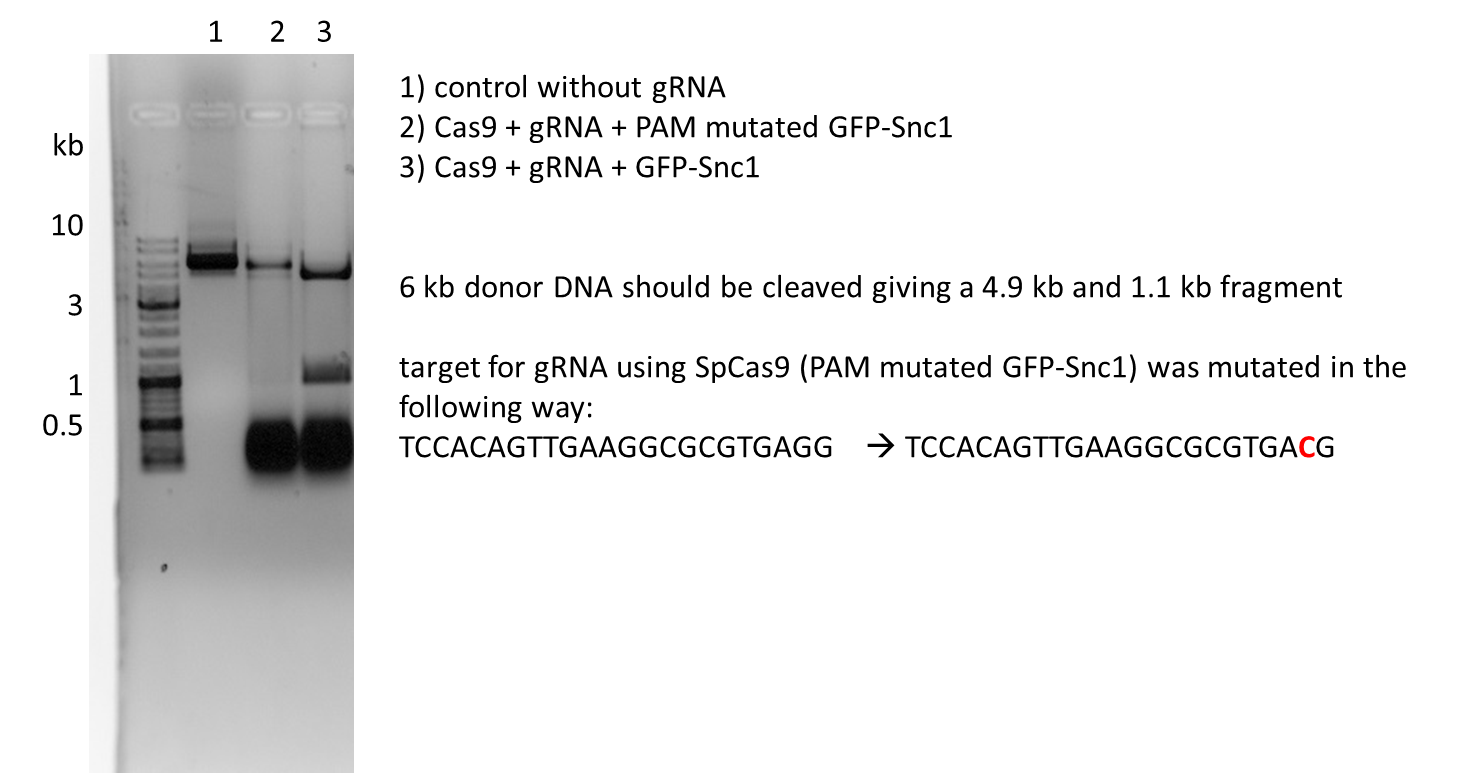

Supplement: Supplementary file 2 — Additional file 2. In vitro cleavage assay of snc1 target site for SpCas9. [file 40694_2019_79_MOESM2_ESM.png]

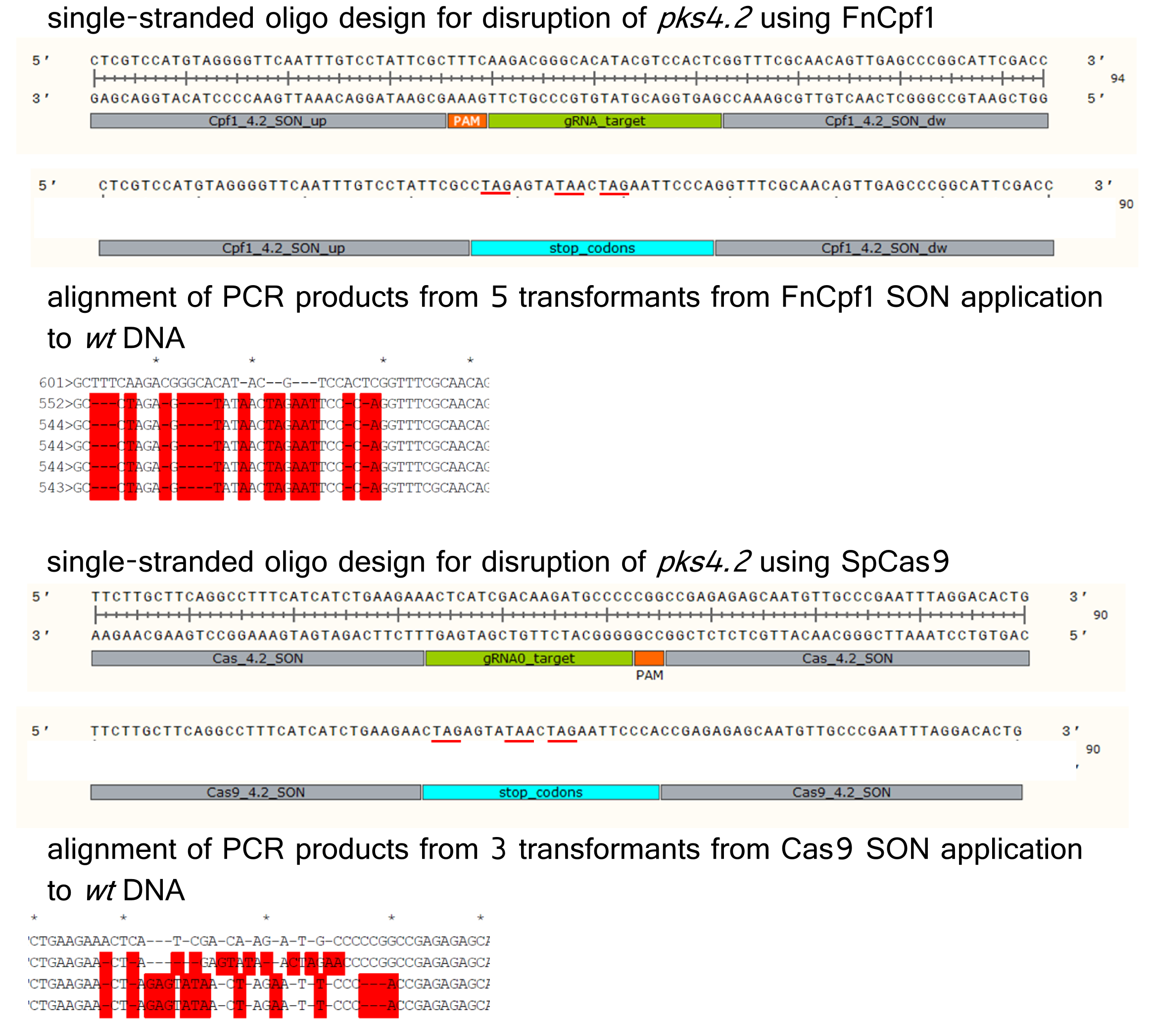

Supplement: Supplementary file 3 — Additional file 3. Application of SNOs using Cpf1 and Cas9. [file 40694_2019_79_MOESM3_ESM.png]
