# Supplementary material for: Practical guidance for the implementation of the CRISPR genome editing tool in filamentous fungi
Source: Fungal Biol Biotechnol. 2019 Oct 17;6:15. doi: 10.1186/s40694-019-0079-4 (PMC6796461; doi:10.1186/s40694-019-0079-4)
Supplement: Supplementary file 4 — Additional file 4. Strains used in this study. [file 40694_2019_79_MOESM4_ESM.docx]

**Additional File 4 – Strains used in this study.**

| **Strain** | **Relevant genotype** | **Reference** |
| --- | --- | --- |
| ATCC42464 | WT | [1] |
| MJK19.4 | Δ*ku80::amds* derivative of ATCC42464 | This study |
| MJK20.2 | *Δku80*, FAA counter selected of MJK19.4 | This study |
| MJK49.6 | *Δku80*, *Δpks4.2*::*amds* (derivative of MJK20.2) | This study |
| MJK52.2 | *Δku80*, *Δpks4.2*::*amds*, *Psnc1*::*GFP*::*snc1* (derivative of MJK20.2) | This study |
| MJK53.1 | *Δku80*, *Δpks4.2*::*amds*, *Psnc1*::*GFP*::*snc1*, *Δalp1* (derivative of MJK20.2) | This study |
| MJK36.5 | *Δku80*, *Δpks4.2*::*amds*, *Psnc1*::*GFP*::*snc1*, *Δalp1, Δptf1* (derivative of MJK20.2) | This study |
| MJK47.3 | *Δku80*, *Δpks4.1*::*amds*, *pks4.2^-^* (derivative of MJK20.2 with SON-mediated premature stop codon in *pks4.2*) | This study |

**Reference**

[1] Berka RM, Grigoriev IV, Otillar R, Salamov A, Grimwood J, Reid I, et al. 2011. Comparative genomic analysis of the thermophilic biomass-degrading fungi *Myceliophthora thermophile* and *Thielavia terrestris*. Nat. Biotechnol. 29: 922-927
