# Supplementary material for: Practical guidance for the implementation of the CRISPR genome editing tool in filamentous fungi
Source: Fungal Biol Biotechnol. 2019 Oct 17;6:15. doi: 10.1186/s40694-019-0079-4 (PMC6796461; doi:10.1186/s40694-019-0079-4)
Supplement: Supplementary file 5 — Additional file 5. Primers and gRNA sequences used in this study. [file 40694_2019_79_MOESM5_ESM.docx]

**Additional File 5. Primers and gRNA sequences used in this study.** Non-homologous sequences which were added due to cloning purposes are depicted in lower case. PAM sequences are highlighted in red.

| **Primers used for constructing Cpf1 plasmids meant for heterologous expression in *Escherichia coli*** | | |
| --- | --- | --- |
| Cloning of FnCpf1 | MTFnCpf1_orf_fw | cgacaagcttgcggccgcATGAGCATCTACCAGGAGTTCG |
| Cloning of FnCpf1 | MTFnCpf1_ORF_rev | tggaggtccggagccgccGTTGTTGCGGTTCTGGACGAAC |
| Cloning of AsCpf1 | MTAsCpf1_orf_fw | cgacaagcttgcggccgcATGACCCAGTTCGAGGGCTTCA |
| Cloning of AsCpf1 | MTAsCpf1_ORF_rev | tggaggtccggagccgccGTTGCGCAGCTCCTGGATGTAG |
| Cloning of pET28a backbone | pET28a_fw | GGCGGCTCCGGACCTCCAAA |
| Cloning of pET28a backbone | pET28a_rev | GCGGCCGCAAGCTTGTCG |
| **Donor DNA construction** | | |
| *amds* amplification | PamdS_p1_fw | GTGTTGCCTCCTGATCCAGT |
| *amds* amplification | amds_P2_rev | CATGGGTTGAGTGGTATGGG |
| *pks4.2*-upstream | pks4.2_5-fw | ccagatcttccggatggctcgagCGGACGTTGTAAAGGGAAAA |
| *pks4.2*-upstream | pks4.2_5_rev | actggatcaggaggcaacacGCTTGAGAAGCCTGCTATCG |
| *pks4.2*-downstream | pks4.2_3_fw | cccataccactcaacccatgAGGGAGGCCCTTATAGACGA |
| *pks4.2*-downstream | pks4.2_3_rev | tgagaatattgtaggagatcttctagaTAGGATGGGCTAGGATGACG |
| *pks4.1*-upstream | pks4.1_5-fw | ccagatcttccggatggctcgagACTTGCCCGTCTTACACAGC |
| *pks4.1*-upstream | pks4.1_5_rev | actggatcaggaggcaacacGTTGAATGTTGCAGTTGGGCA |
| *pks4.1*-downstream | pks4.1_3_fw | cccataccactcaacccatgGATGGATGGATGGACGTCAGC |
| *pks4.1*-downstream | pks4.1_3_rev | tgagaatattgtaggagatcttctagaTACAACGCCATGGTTAGCAA |
| *snc1*-upstream | PSnc1_XhoIpJet_fw | ccagatcttccggatggctcgagGCGGAAGATCCAATGTCTGT |
| *snc1*-upstream | PSnc1_P2_gfp_rev | agctcctcgcccttgctcaccatTTTGACAATCGCAGACGACG |
| *egfp* amplification | GFP_P1_fw | atgGTGAGCAAGGGCGAGGAGCT |
| *egfp* amplification | GFP_P2_5GA_rev | ggcaccggcgccagcaccagcgccggcaccCTTGTACAGCTCGTCCATGC |
| *snc1* ORF amplification | Snc1_orf_5_GA_fw | ggtgccggcgctggtgctggcgccggtgccATGTCCACGTCGCCCTACGA |
| *snc1* ORF amplification | Snc1_orf_XbaIpJet_rev | tgagaatattgtaggagatcttctagaATTAATTGCCCAAGGGAACC |
| *alp1*-upstream | Alp1_5_fw | ccagatcttccggatggctcgagCCATTGCTGCCCTTGCTTTG |
| *alp1*-upstream | Alp1_5_rev | cgatggataattgtgccgtgTTGGGGTGTTGGAGACGATG |
| *alp1*-downstream | Alp1_3_fw | cacggcacaattatccatcgAGCACTTCCTGCACCTCTTC |
| *alp1*-downstream | Alp1_3_rev | tgagaatattgtaggagatcttctagaTAAGGGTCGGACACACTCAG |
| **Construction of sgRNA expressing plasmids** | | |
| *pks4.2* & *snc1* gRNA | pgR_4.2snc_plas_fw | AGATAAGACGGGCACATACGTCCAAATTTCTACTGTTGTAGATGGCTTCGGCTGCGAACCCCC |
| *pks4.2* & *snc1* gRNA | pgR_4.2snc_plas_rev | AAACGGGGGTTCGCAGCCGAAGCCATCTACAACAGTAGAAATTTGGACGTATGTGCCCGTCTT |
| *alp1* gRNA | pgR_alp1_plas_fw | AGATCCGGCAAGCTCGAGGATGAC |
| *alp1* gRNA | pgR_alp1_plas_re | AAACGTCATCCTCGAGCTTGCCGG |
| **Target locus** | **Nuclease** | **Target sequence with PAM** |
| *pks4.2* | Cpf1 | TTTCAAGACGGGCACATACGTCCACTC |
|  | Cas9 | ACTCATCGACAAGATGCCCCCGG |
| *pks4.1* | Cpf1 | TTTGGCGACCAAGCGGTCCCTTACGCG |
|  | Cas9 | TATCGTCTTTGGCGACCAAGCGG |
| *snc1* | Cpf1 | TTTGGGCTTCGGCTGCGAACCCCCATC |
|  | Cas9 | TCCACAGTTGAAGGCGCGTGAGG |
| *alp1* | Cpf1 | TTTGCCGGCAAGCTCGAGGATGAC |
|  | Cas9 | CGACCTGGCCAGCGCGAGCAGTCAGG |
| **Diagnostic PCR & Southern blot probe** | | |
| *ku80,* 5’ probe | Dku80_5-fw | ccagatcttccggatggctcgagAAATCTCGAGCGACAGCGA |
| *ku80,* 5’ probe | Dku80_5-rev | actggatcaggaggcaacacTCGAGATTCCCGACCGAACG |
| *ku80,* 3’ probe | Dku80_3-fw | cccataccactcaacccatgTGATTCGGTCGTGTAGGCTT |
| *ku80,* 3’ probe | Dku80_3-rev | tgagaatattgtaggagatcttctagaAACTGACGCTCGACTGG |
| *pks4.2 5’* integration | pks4.2_5_fws | CCTGCTGGCTACTTGGAGAC |
| *pks4.1 5’* integration | pks4.1_5_fws | GCATGTAGCTTAGCCTTGCC |
| *amds* maker for 5’ integration | Tan122 | CCACTTCTGGAGATGCGTGTCC |
| *pks4.2 5’* integration (no *amdS* marker) | pks4.2_3_rev | tgagaatattgtaggagatcttctagaTAGGATGGGCTAGGATGACG |
| *snc15’*  integration | Psnc1_5_fws | TGGTTGCTGCTGTTGGATAG |
| *Snc1 5’* integration | GFP_P2_5GA_rev | ggcaccggcgccagcaccagcgccggcaccCTTGTACAGCTCGTCCATGC |
| *al1* 5’ integration (no amds marker) | Haf3703 | CACCACCCCTCCATTTCTTG |
| *al1* 5’ integration (no amds marker) | Alp1_3_rev | tgagaatattgtaggagatcttctagaTAAGGGTCGGACACACTCAG |
| **Construction of sgRNA template for *in vitro* transcription for RNP application** | | |
| *ku80* gRNA for FnCpf1 | gRNA_ku80_1_fw | ATGTAATACGACTCACTATAGGTAATTTCTACTGTTGTAGATTCTATGCGCTATGTCTGGGACAA |
| *ku80* gRNA for FnCpf1 | gRNA_ku80_1_rev | TTGTCCCAGACATAGCGCATAGAATCTACAACAGTAGAAATTACCTATAGTGAGTCGTATTACAT |
| *pks4.1* gRNA for FnCpf1 | gRN_pks4_1_fn_f | ATGTAATACGACTCACTATAGGTAATTTCTACTGTTGTAGATGCGACCAAGCGGTCCCTTACGCG |
| *pks4.1* gRNA for FnCpf1 | gRN_pks4_1_fn_r | CGCGTAAGGGACCGCTTGGTCGCATCTACAACAGTAGAAATTACCTATAGTGAGTCGTATTACAT |
| *pks4.2* gRNA for FnCpf1 | gRN_pks4_2_fn_f | ATGTAATACGACTCACTATAGGTAATTTCTACTGTTGTAGATTCATTGGACTTTGCTCCGGGCTT |
| *pks4.2* gRNA for FnCpf1 | gRN_pks4_2_fn_r | AAGCCCGGAGCAAAGTCCAATGAATCTACAACAGTAGAAATTACCTATAGTGAGTCGTATTACAT |
| *pks4.1* gRNA for AsCpf1 | gRN_pks4_1_as_f | ATGTAATACGACTCACTATAGGTAATTTCTACTCTTGTAGATGCGACCAAGCGGTCCCTTACGCG |
| *pks4.1* gRNA for AsCpf1 | gRN_pks4_1_as_f | CGCGTAAGGGACCGCTTGGTCGCATCTACAAGAGTAGAAATTACCTATAGTGAGTCGTATTACAT |
| *pks4.2* gRNA for AsCpf1 | gRN_pks4_2_as_f | ATGTAATACGACTCACTATAGGTAATTTCTACTCTTGTAGATTCATTGGACTTTGCTCCGGGCTT |
| *pks4.2* gRNA for AsCpf1 | gRN_pks4_2_as_f | AAGCCCGGAGCAAAGTCCAATGAATCTACAAGAGTAGAAATTACCTATAGTGAGTCGTATTACAT |
| SpCas9 gRNA standard rev | sgRNAstd_rev | AGCACCGACTCGGTGCCACTTTTTCAAGTTGATAACGGACTAGCCTTATTTTAACTTGCTATTTCTAGCTCTAAAAC |
| *ku80* gRNA for Cas9 | p_ku80_Cas_g | ATGTAATACGACTCACTATAGGGAGCACATCTCTGTCCTTCAGTTTTAGAGCTAGAAATAGCAAGT |
| *pks4.1* gRNA for Cas9 | p_pks4_1_cas | ATGTAATACGACTCACTATAGGTATCGTCTTTGGCGACCAAGGTTTTAGAGCTAGAAATAGCAAGT |
| *pks4.2* gRNA for Cas9 | p_pks4_2_cas | ATGTAATACGACTCACTATAGGACTCATCGACAAGATGCCCCGTTTTAGAGCTAGAAATAGCAAGT |
| *snc1* gRNA for Cas9 | Psnc1_cas_f | ATGTAATACGACTCACTATAGGTCCACAGTTGAAGGCGCGTGGTTTTAGAGCTAGAAATAGCAAGT |
| *alp1* gRNA for Cas9 | Alp1 Cas | ATGTAATACGACTCACTATAGGCGACCTGGCCAGCGCGAGCAGTCGTTTTAGAGCTAGAAATAGCAAGT |
| FnCpf1 gRNA standard for | pT7_FnCpf1_fw | ATGTAATACGACTCACTATAGGTAATTTCTACTGTTGTAGAT |
| *snc1* gRNA for FnCpf1 | Snc1_1FnR | GATGGGGGTTCGCAGCCGAAGCCATCTACAACAGTAGAAATTA |
| *alp1* gRNA for FnCpf1 | Alp1 Fn | GTCATCCTCGAGCTTGCCGGATCTACAACAGTAGAAATTA |
| AsCpf1 gRNA standard for | pT7_ASCpf1_fw | ATGTAATACGACTCACTATAGGTAATTTCTACTCTTGTAGAT |
| *snc1* gRNA for AsCpf1 | Snc1_1AsR | GATGGGGGTTCGCAGCCGAAGCCATCTACAAGAGTAGAAATTA |
| *alp1* gRNA for AsCpf1 | Alp1 As | GTCATCCTCGAGCTTGCCGGATCTACAAGAGTAGAAATTA |
| **Target locus** | **Nuclease** | **Target sequence with PAM** |
| *ku80* | FnCpf1 | TTCTCTATGCGCTATGTCTGGGACAA |
|  | Cas9 | GAGCACATCTCTGTCCTTCAAGG |
| *pks4.2* | Cpf1 | TTTCAAGACGGGCACATACGTCCACTC |
|  | Cas9 | ACTCATCGACAAGATGCCCCCGG |
| *pks4.1* | Cpf1 | TTTGGCGACCAAGCGGTCCCTTACGCG |
|  | Cas9 | TATCGTCTTTGGCGACCAAGCGG |
| *snc1* | Cpf1 | TTTGGGCTTCGGCTGCGAACCCCCATC |
|  | Cas9 | TCCACAGTTGAAGGCGCGTGAGG |
| *alp1* | Cpf1 | TTTGCCGGCAAGCTCGAGGATGAC |
|  | Cas9 | CGACCTGGCCAGCGCGAGCAGTCAGG |

Note that primers as well as sgRNAs targeting *ptf1* are not shown due to patent issues. However, they can be requested from the authors after clearance and upon reasonable request.
