# Supplementary material for: Practical guidance for the implementation of the CRISPR genome editing tool in filamentous fungi
Source: Fungal Biol Biotechnol. 2019 Oct 17;6:15. doi: 10.1186/s40694-019-0079-4 (PMC6796461; doi:10.1186/s40694-019-0079-4)
Supplement: Supplementary file 6 — Additional file 6. Plasmids used in this study. [file 40694_2019_79_MOESM6_ESM.docx]

**Additional File 6 – Plasmids used in this study.**

| **Name** | **Description** | **Reference** |
| --- | --- | --- |
| pET28a/Cas9-Cys | Expression of N-terminal His-tag fused human codon optimized Ca9 nuclease having C-terminal Cysteine | Addgene (#53261) |
| pMJK16.1 | Expression of N-terminal His-tag fused *T. thermophilus* codon optimized FnCpf1 nuclease | This study |
| pMJK17.1 | Expression of N-terminal His-tag fused *T. thermophilus* codon optimized AsCpf1 nuclease | This study |
| pMJK21.1 | Deletion cassette of *pks4.2* with *amdS* selection marker | This study |
| pMJK13.1 | Expression of *gfp::snc1*, Cpf1 target PAM sequence was mutated (TTTG --> TgTG) | This study |
| pMJK22.19 | Deletion cassette of *pks4.1* with amdS selection marker | This study |
| pMJK26.1 | Deletion cassette of *alp1* without selection marker | This study |
| pMJK27.1 | Deletion cassette of *ptf1* without selection marker | This study |
